# Transcriptomic analysis of the response of *Avena sativa* to *Bacillus amyloliquefaciens* DGL1

**DOI:** 10.3389/fmicb.2024.1321989

**Published:** 2024-04-03

**Authors:** Xue Yang, Yongli Xie, Tian Wang, Youming Qiao, Junxi Li, Lingling Wu, Ying Gao

**Affiliations:** ^1^College of Agriculture and Animal Husbandry, Qinghai University, Xining, Qinghai, China; ^2^Key Laboratory of Use of Forage Germplasm Resources on Tibetan Plateau of Qinghai Province, Xining, Qinghai, China; ^3^State Key Laboratory of Plateau Ecology and Agriculture of Qinghai University Xining, Xining, Qinghai, China

**Keywords:** *Bacillus amyloliquefaciens*, extreme habitats, growth promotion, oats, transcriptome sequencing

## Abstract

**Introduction:**

*Bacillus amyloliquefaciens* DGL1, isolated from the arid sandy areas in Dagler, Qinghai Province, China, promotes the growth of *Avena sativa* variety “Qing Yan 1”.

**Methods:**

To elucidate the transcriptomic changes in the oat root system following interaction with DGL1 and to reveal the molecular mechanism by which DGL1 promotes oat growth, treatment and control groups of oat roots at 2, 4, 8, and 12 h after inoculation with a suspension of strain DGL1 were analyzed using Illumina high-throughput transcriptome sequencing technology. The differentially expressed genes were determined through Gene Ontology (GO) and Kyoto Encyclopedia of Genes and Genomes (KEGG) pathway enrichment analyses, and the metabolic pathways and key genes were analyzed.

**Results:**

The results showed that 7874, 13,392, 13,169, and 19,026 differentially expressed genes were significantly enriched in the glycolysis/gluconeogenesis pathway, amino acid metabolism, nitrogen metabolism, plant hormone signal transduction, and other related metabolic pathways in the oat roots at 2, 4, 8, and 12 h after inoculation with a DGL1 suspension. The GO and KEGG enrichment analyses revealed that the genes encoding plasma membrane ATPase, phosphoglycerate kinase gene *PGK*, ammonium transporter protein gene *AMT*, cellulose synthase gene *CSLF6*, and growth hormone response family gene *IAA18* were significantly upregulated.

**Discussion:**

It is hypothesized that the pro-growth mechanism of strain DGL1 in oats is the result of the coordination of multiple pathways through the promotion of oat energy metabolism, phytohormone signaling, secondary metabolite synthesis, and amino acid metabolism.

## 1 Introduction

*Avena sativa* is an annual herb with a well-developed fibrous root system covering a wide area. It is resistant to cold, salinity, and drought, and it can effectively reduce soil erosion and fix soil (Łabanowska et al., 2016; Liu et al., 2017; Guan et al., 2018). Oats have tender stalks and good palatability, are rich in protein and microbiotin, and are preferred by livestock, such as horses, cattle, and sheep, making them a high-quality forage feed. In addition, oats are rich in bioactive compounds, such as β-glucan, phenolic acid, sterol, oat glycoside, and anisamide, making them a natural health food (Chen et al., 2018; Nan et al., 2018). Therefore, oats have both economic and ecological value. Oats require nitrogen, phosphorus, potassium, and trace elements, such as calcium, magnesium, and zinc, for growth in order to maintain high yields, however excessive or incorrect fertilization will lead to changes in the physical, chemical, and microbiological characteristics of the soil and the leaching or evaporation of fertilizer into the atmosphere and waterbodies, resulting in nutrient deficits and environmental pollution (Mustafa, 2011).

Biofertilizer application is an environmentally friendly biological strategy in plant cultivation, with beneficial *Bacillus* activating host plant immunity without negatively impacting the host or the environment (Radhakrishnan et al., 2017). *Bacillus* can increase the availability of nutrients in the soil by fixing nitrogen in the air into ionic form and by secreting organic acids to convert phosphate into soluble phosphorus for plant uptake and use (Choudhary and Johri, 2008; Ortiz and Sansinenea, 2018). It can also secrete the plant growth hormones indoleacetic acid (IAA), cytokinin, and gibberellin to induce plant cell division and root differentiation, elongation, and growth, produce the volatile organic compounds 3-hydroxy-2-butanone and 2,3-butanediol, and establish beneficial relationships with host plants, thereby increasing the chlorophyll and proline contents and crop yields, offering new opportunities for sustainable agricultural development (Elshaghabee et al., 2017; Saeed et al., 2021).

Transcriptome sequencing analysis or RNA sequencing (RNA-Seq) is a novel high-throughput technology based on second-generation sequencing that is used to determine gene expression under specific tissue and physiological conditions. It reflects expression levels and plays an important role in the discovery and analysis of new transcripts, the functional analysis of expressed genes, and the assessment of metabolic pathways in biological processes (Guo et al., 2020). It has been used in many fields such as agriculture, medicine, and industry. After a preliminary laboratory study, *Bacillus amyloliquefaciens* strain DGL1, isolated from the arid and sandy areas of the Qinghai–Tibet Plateau at an altitude of 3,010 m, was found to have good cellulose degradation and nitrogen fixation activities and to significantly inhibit the growth of *Fusarium oxysporum, Fusarium acuminatum*, and *Fusarium graminearum* (Yang et al., 2022). It also showed significant pro-life activity in *Avena sativa, Poa crymophila*, and *Festuca rubra*. DGL1 has gene clusters encoding the lipopeptide repressor fengycin and surfactin proteins and can reach 100% homology with the gene cluster encoding fengycin, as well as the key genes *yhcX, dhaS*, and *YsnE* for growth hormone synthesis and *alsR* for the synthesis of the plant stress resistance enhancing substance 2,3-butanediol (Yang et al., 2020). To investigate the molecular mechanism by which DGL1 promotes oat growth, this study was conducted by establishing a mutualistic system between strain DGL1 and oats, and after 2, 4, 8, and 12 h of mutualism, RNA was extracted from oat roots. RNA-Seq was performed on the Illumina NovaSeq 6000 sequencing platform to help us better understand the physiological and energy metabolic processes that *Bacillus* induces in oat roots and the complex network of signal transduction in plants. In addition, the expression of key genes in *Bacillus* DGL1-induced oat-related signaling and metabolic pathways was analyzed through Gene Ontology (GO) enrichment analysis and Kyoto Encyclopedia of Genes and Genomes (KEGG) pathway enrichment analysis. Four differentially expressed growth-promoting functional genes in the interaction group were screened to investigate the mechanism of DGL1-induced oat growth in extreme habitats.

## 2 Materials and methods

### 2.1 Materials

The plant material was *Avena sativa* variety “Qing Yan 1,” which was provided by the Key Laboratory of the Use of Forage Germplasm Resources on the Tibetan Plateau in Qinghai Province.

The test strain, *Bacillus amyloliquefaciens* DGL1, was stored in an ultra-low temperature refrigerator at −80°C (Yingcan Co., Beijing, China).

### 2.2 Preparation of a *Bacillus* suspension

Growth curve, The DGL1 was cultured in LB liquid medium at 37°C, 200 rpm, and the absorbance was measured at 0, 2, 4, 6, 8, 10, 12, 14, 16, 18, 20, 22 and 24 h, repeated three times, and the growth curves were prepared using the software origin.

Bacterial solution, An inoculating loop was dipped into the DGL1 stored at −80°C and scribed on a Luria Broth (LB*)* solid plate, and the plate was incubated overnight at 37°C. After a single colony had grown on the LB plate, the single colony was used to inoculate a triangular flask containing 20 mL of LB liquid medium, and the flask was incubated at 37°C for 12 h at 200 rpm. The culture was centrifuged to extract the bacterium and then suspended in sterile water. The concentration of the bacterial solution was adjusted to 10^6^ cfu·mL^−1^ to prepare a *Bacillus* suspension (Xie et al., 2017).

### 2.3 Interaction of oats with a DGL1 bacterial suspension

Oat grass seeds with intact seed coats of uniform size were selected, disinfected in 20% sodium hypochlorite solution for 30 min, rinsed 5 times with sterile water, and sown in cavity pots (30 plants/pot) with sterilized nutrient soil. The pots were incubated at 25°C with a photoperiod of 16/8 h, and 20 mL of distilled water was added every 3 d. The oat seedlings were removed from the cavity pots after 12 d of incubation and divided into 5 groups. The roots were washed several times with sterile water, and filter paper was used to remove water from the roots. Four of the groups were completely immersed in the DGL1 suspension at a cell concentration of 1 × 10^6^ cfu·mL^−1^ to establish an interaction system between oats and the DGL1 bacterial suspension, and the other group was immersed in sterile water for 2, 4, 8, and 12 h.

### 2.4 RNA extraction from oat roots and sequencing library construction

The oats were removed from the suspension at 2, 4, 8, and 12 h. The roots were quickly cut with sterilized scissors, wrapped in tin foil, and stored in liquid nitrogen at −80°C in an ultra-low temperature refrigerator for later use. The samples were entrusted to the Shanghai Meiji Pharmaceutical and Biological Company (Shanghai, China) for RNA extraction, sequencing, and library construction (Illumina, San Diego, CA, USA). The steps of the transcriptome sequencing experiment were as follows: total RNA was extracted from oat roots (5 groups of samples with 3 replicates per group for 15 groups in total) (Tiangen Biotech Co., Ltd., Beijing, China). RNA quality was checked in the 15 extracted samples; the mRNA was isolated using magnetic beads; the isolated complete mRNA was fragmented; the mRNA was reverse transcribed into cDNA under the action of reverse transcriptase, followed by the synthesis of duplexes to form a stable double-stranded structure; and machine sequencing was conducted (Xu et al., 2022).

### 2.5 Transcriptome data analysis

#### 2.5.1 Analysis of differentially expressed genes (DEGs)

Sequencing data were compared with oat reference genome sequences using TopHat2 software. Gene expression was quantitatively analyzed via Fragments per kilobase of transcript per million fragments mapped (FPKM) algorithm. Genes with differential expression between comparison groups were statistically analyzed using DESeq2 software, with default parameters: p-adjust <0.05, |log2FC| ≥ 1. The oat group with sterile water was used as a control, and SeqPrep software was used for sequencing data quality control. EdgeR software was used to draw the volcano map of DEGs, python software was used for Principal component analysis (PCA).

#### 2.5.2 GO functional analysis and KEGG enrichment analysis

Statistical analysis of genes with expression differences between comparative groups was performed using DESeq2 software with default parameters: p-adjust <0.05, |log2FC| ≥ 1. The oat group with sterile water was used as a control to identify genes with expression changes after the oat was inoculated with a DGL1 bacterial suspension. TopHat2 software was used to compare the sequencing data with oat reference genome sequences. Goatools software was used for GO enrichment analysis (Gene, 2010) to analyze the gene functions of oats after interaction with strain DGL1. The KEGG (Kanehisa and Goto, 2011) database was used to enrich the differential genes and analyze the changes in gene expression, and this study mainly analyzed probiotic-related differential genes.

### 2.6 Real-time fluorescence quantitative PCR (qRT-PCR) analysis

To validate the RNA-Seq data, the *PSKS1* gene of oat was used as an internal reference gene, and the genes *PGK, AMT*, and *Hsp20* were selected for qRT-PCR analysis in each of the four reciprocal treatment groups. The qRT-PCR was performed using the following cycling conditions: 95°C for 30 s, 40 cycles at 95°C for 15 s and 60°C for 30 s, followed by an extension. Each PCR analysis was repeated three times, and the relative expression of the genes was calculated using the 2^−Δ*ΔCt*^ relative quantification method. Statistically significant differences were determined using Student–Newman–Keuls test (*p* < 0.05).

## 3 Results and analysis

### 3.1 Transcriptome sequencing data statistics

In this study, the Illumina sequencing platform was used to complete transcriptome sequencing, and the sequence data of the 15 samples obtained were evaluated for base accuracy using the Q20 and Q30 quality values. A total of 111.68 Gb of clean data were obtained from the transcriptome analysis of 15 samples in this study, and the clean data of all 15 samples were above 6.33 Gb in size. The Q30 score was above 95.43%, indicating a low sequencing error rate and good sequencing quality. SeqPrep software was used to remove junction sequences and low-quality and excessive N reads, and TopHat2 software was used to compare each of the 15 samples with the oat reference genome sequence. The comparison rate was >78.92%, with the GC content ranging from 53 to 57%.

### 3.2 Analysis of DEGs

**Figure 1:**
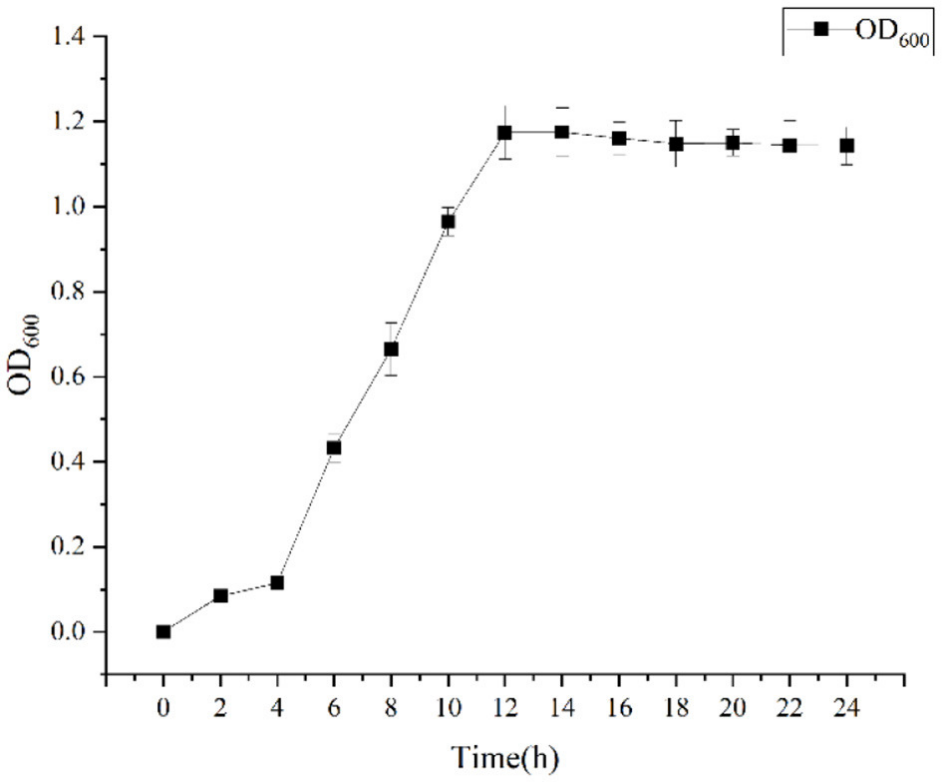
The growth curve of DGL1.

**Figure 2:**
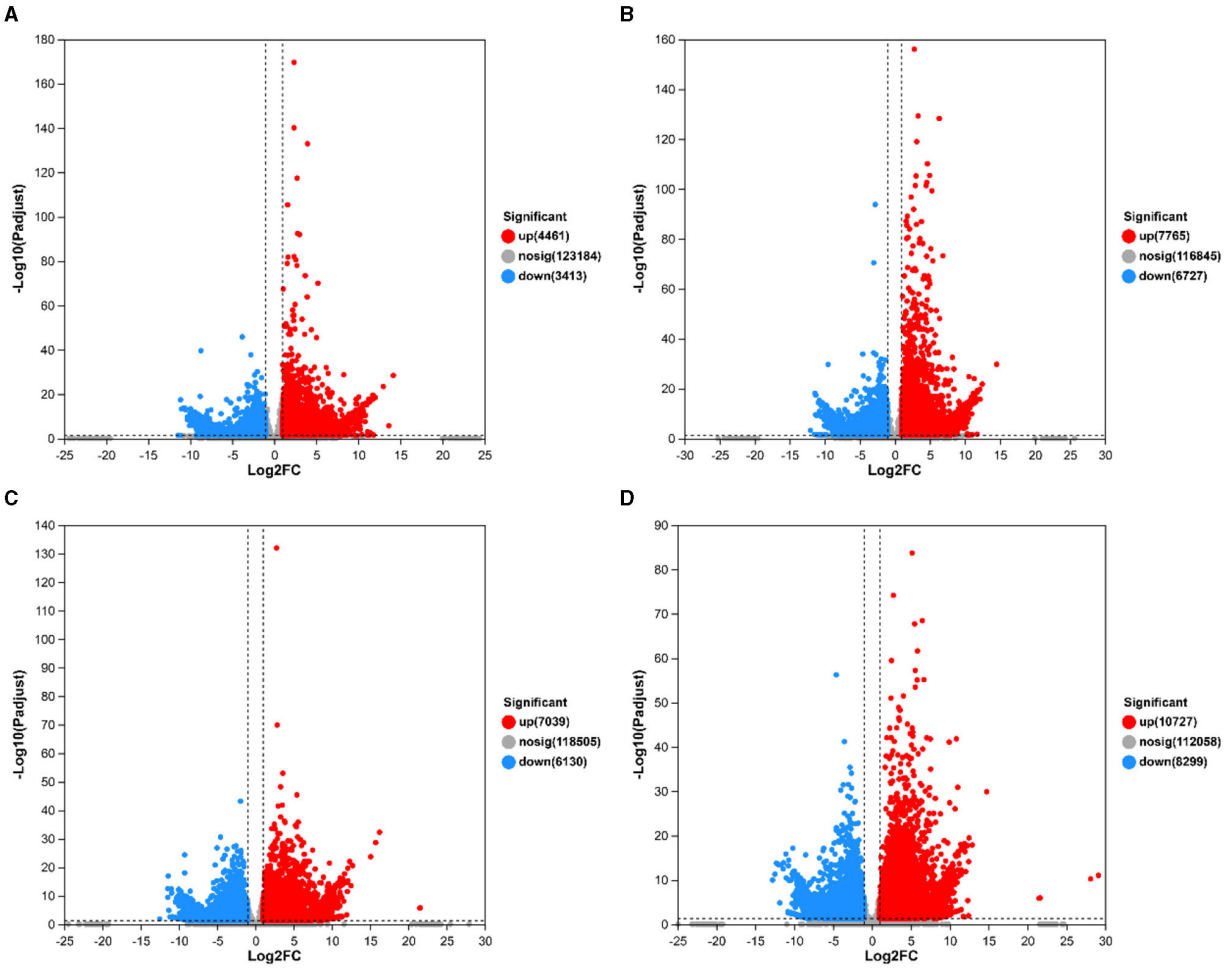
Volcano map of differentially expressed genes. The horizontal coordinates represent the fold difference in gene expression, the vertical coordinates represent the statistical test value of the difference in gene expression change, i.e., *p*-value, and gray circles, red circles, and green circles represented genes with no significant changes, upregulated genes, and downregulated genes, respectively. **(A)** CKa_vs_a2.volcano. **(B)** CKa_vs_a4.volcano. **(C)** CKa_vs_a8.volcano. **(D)** CKa_vs_a12.volcano.

Growth was monitored while cultured in an LB medium to investigate time-related growth changes in DGL1. DGL1 reached the logarithmic growth phase after 4 h and reached a plateau around 12 h (Figure 1). Statistical analysis of the DEGs between comparative groups was performed using DESeq2 software, with the sterile water interaction group as the control, under default parameters: *p*-adjust < 0.05, |log2FC| ≥ 1. The volcano map of DEGs is shown in Figure 2, the four treatment groups a2 (2 h interaction group), a4 (4 h interaction group), a8 (8 h interaction group), and a12 (12 h interaction group) were compared with the control group (CKa). The results showed that 7874, 13392, 13,169, and 19,026 DEGs were identified at 2, 4, 8, and 12 h after inoculation, respectively. A total of 4,461, 7,765, 7,039, and 10,727 genes were upregulated, and 3,413, 6,727, 6,130, and 8,299 genes were downregulated at 2, 4, 8, and 12 h after inoculation, respectively (Figure 2).

**Figure 3:**
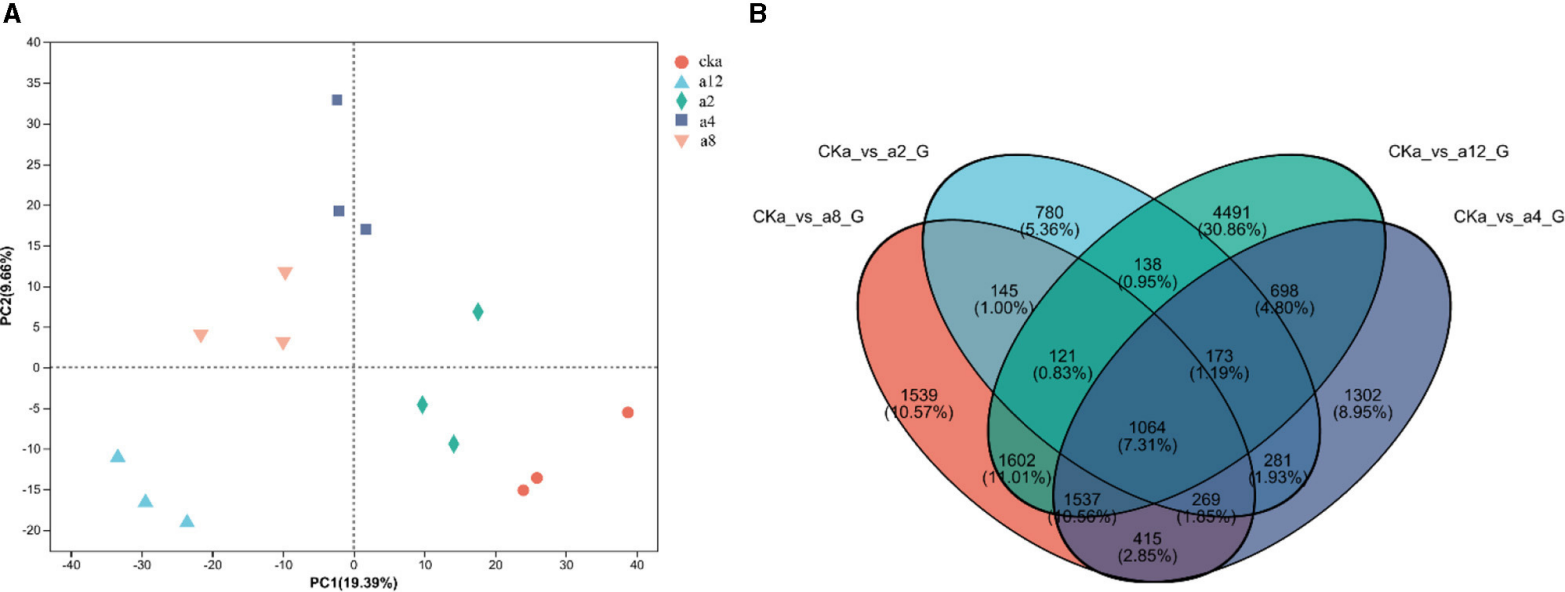
**(A)** The results of PCA analysis. **(B)** Venn diagram of DEGs in four comparison groups.

As shown in Figure 3, The twelve samples were separated from each other in the PCA plot, and three replicates of each sample were tightly grouped, indicating that the sequencing data was highly reproducible. Meanwhile the difference between treatment groups was obvious in the PCA plot. These showed that DGL1 had a significant effect on the gene expression of *Avena sativa* (Figure 3). The Venn diagram of DEGs is shown in Figure 3. A total of 14,455 genes were shared by the four comparison groups, and 780, 1,302, 1,539, and 4,491 DEGs were expressed only in a2, a4, a8, and a12, respectively.

### 3.3 GO enrichment analysis of DEGs

The software Goatools was used to identify the DEGs in the four treatment groups and to obtain information on the biological processes, molecular functions, and cellular components in which the DEGs were involved in the 2, 4, 8, and 12 h reciprocal groups.

**Figure 4:**
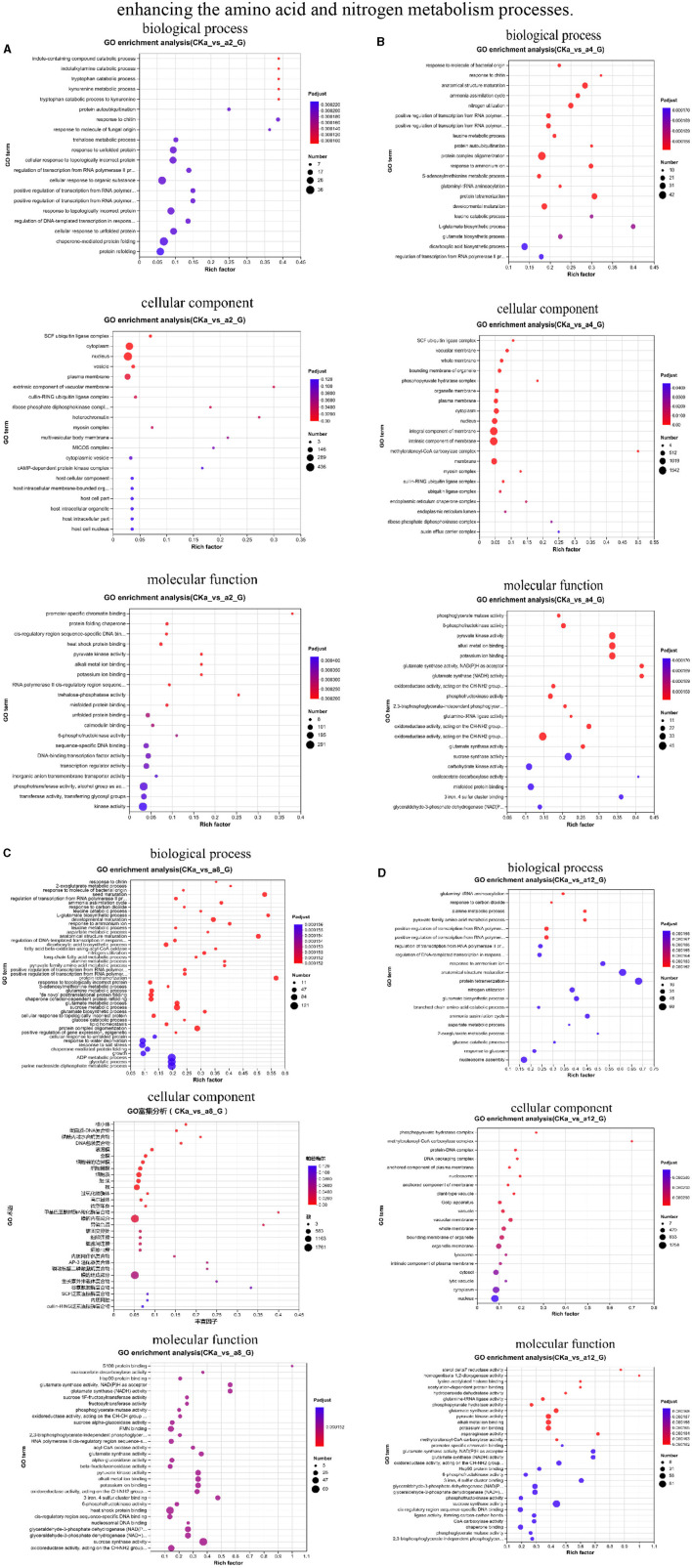
Classification of gene ontology (GO) function. **(A)** 2 h difference comparison group GO functional classification, **(B)** 4 h difference comparison group GO functional classification, **(C)** 8 h difference comparison group GO functional classification, **(D)** 12 h difference comparison group GO functional classification. The vertical axis indicates the GO term, the horizontal axis indicates the Rich factor, the size of the dots indicates the number of genes/transcripts in this GO term, and the color of the dots corresponds to different *P*-adjust ranges.

In the 2 h interaction, the DEGs were significantly enriched in biological processes related to plant cell metabolism, such as alginate metabolism and response to nitrogen compounds, in cellular components related to the nucleus, vesicles, and plasma membrane; in other processes related to the formation of plant cells; and in molecular functions related to heat shock protein (Hsp) binding, pyruvate kinase activity, and 6-phosphate fructokinase activity (Figure 4A).

During the 4 h interaction, the genes were significantly enriched in processes related to the ammonia assimilation cycle, nitrogen utilization, and response to ammonium ions in biological processes. In cellular processes, the genes were significantly enriched in processes related to cellular components, such as vesicle membranes, organelle membranes, and plasma membranes. In the molecular functional processes, the genes were significantly enriched in processes related to 6-phosphofructokinase activity and pyruvate kinase activity (Figure 4B).

In the 8 h interaction, the genes were significantly enriched in sucrose metabolism, response to nitrogen compounds, and glycolysis in biological processes. In cellular processes, the genes were significantly enriched in vesicle membranes, cytoplasm, and organelle membranes. In the molecular function processes, the genes were significantly enriched in glutamate synthase activity and pyruvate kinase activity (Figure 4C).

In the 12 h interaction, the genes were significantly enriched in biological processes, such as alanine metabolism, response to ammonium ions, and other metabolic processes related to plant growth and development and stress. In cellular components, the genes were significantly enriched in processes related to plant cells, organelles, and membranes, such as the plasma membrane and vesicles. In molecular functions, the genes were significantly enriched in processes related to plant catalytic activity, such as glutamate synthase activity, pyruvate kinase activity, and sucrose synthase activity (Figure 4D).

In summary, DGL1 interacted with the oat roots, mainly by affecting the growth, metabolism, and glycolysis of oat cell tissues and other metabolic pathways to regulate the growth and development of oats. At 2 h of interaction, the DEGs were significantly enriched in the process of algal sugar metabolism, where algal sugar was catalyzed by enzymes to form glucose and fructose phosphate, providing energy materials for plant growth and metabolism. At 4 h of interaction, the DEGs were significantly enriched in metabolic pathways related to plant nutrient uptake, indicating that DGL1 could induce the uptake and utilization of nitrogen elements in oats and promote the formation of nitrogenous organic substances, such as amino acids and proteins. At 8 h of interaction, the DEGs were significantly enriched in processes related to glutamate synthase activity and pyruvate kinase activity; a key enzyme in ammonium assimilation involved in the primary uptake of nitrogen and the reabsorption of ammonia released by photorespiration (Palmgren and Morsomme, 2020) and the last rate-limiting enzyme in the glycolytic pathway capable of generating pyruvate and ATP through the catalytic action of phosphoenolpyruvate (PEP) (Rojas-Pirela et al., 2003), respectively. Therefore, DGL1 may facilitate the process of ammonium assimilation and the glycolytic pathway to provide energy and nutrients for oat growth and development. At 12 h of interaction, the DEGs were significantly enriched in amino acid metabolism, nitrogen metabolism, and cell and organelle formation processes; thus, it is possible that DGL1 could increase the protein content and oat yield by enhancing the amino acid and nitrogen metabolism processes.

### 3.4 KEGG enrichment analysis

**Figure 5:**
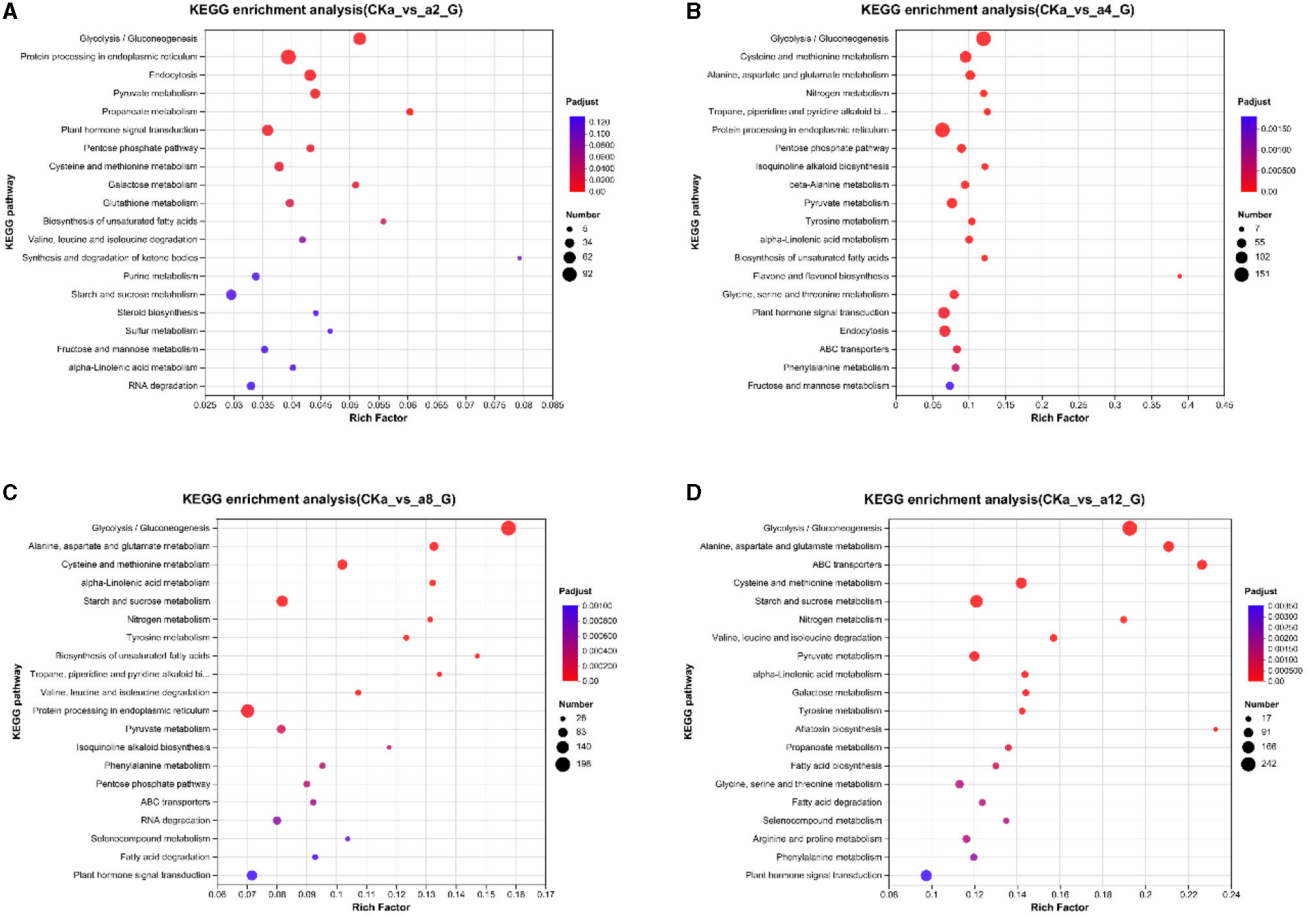
Enriched kyoto encyclopedia of genes and genomes (KEGG) pathways. **(A)** 2 h difference comparison group KEGG functional classification, **(B)** 4 h difference comparison group KEGG functional classification, **(C)** 8 h difference comparison group KEGG functional classification, **(D)** 12 h difference comparison group KEGG functional classification. The vertical axis represents the name of the pathway, the horizontal axis represents the Rich factor, the size of the dots indicates the number of genes in this pathway, and the color of the dots corresponds to different *P*-adjust ranges.

To further investigate the molecular mechanism of oat growth promotion by strain DGL1, the differential genes of the four treatment groups were enriched in the KEGG database. The results showed that the DEGs were significantly enriched in the glycolysis/gluconeogenesis pathway, the protein processing metabolic pathway in the endoplasmic reticulum, the endocytosis metabolic pathway, the pyruvate metabolic pathway, and the phytohormone signaling pathway after 2 h of interaction (Figure 5A). At 4 h of interaction, the DEGs were significantly enriched in the glycolysis/gluconeogenesis metabolic pathway, the cysteine and methionine metabolic pathway, the alanine, aspartate and glutamate metabolic pathways, the nitrogen metabolic pathway, and the protein processing pathways in the endoplasmic reticulum (Figure 5B). At 8 h of interaction, the DEGs were significantly enriched in the glycolytic/gluconeogenic metabolic pathways; alanine, aspartate and glutamate metabolic pathways; cysteine and methionine metabolic pathways; starch and sucrose metabolic pathways; and nitrogen metabolism (Figure 5C). At 12 h of interaction, the DEGs were significantly enriched in the glycolysis/gluconeogenesis pathway; alanine, aspartate and glutamate metabolic pathway; ABC transport metabolic pathway; cysteine and methionine metabolic pathway; and starch and sucrose metabolic pathway (Figure 5D).

Under the induction of strain DGL1, the metabolic pathways of the oat root tissues underwent significant changes and were mainly enriched in glycolysis/gluconeogenesis pathways, amino acid metabolism, nitrogen metabolism, and other related pathways, which induced the synthesis of oat proteins and sugars and promoted growth and development. At 12 h of interaction, the genes were also significantly enriched in the ABC transport metabolic pathway, suggesting that DGL1 could promote the transport process of oat flavonoids, indole acetic acid, benzene propane, and other substances, thus increasing the accumulation of secondary metabolites.

### 3.5 Analysis of relevant probiotic genes

#### 3.5.1 Energy metabolism

Plants maintain their normal life activities by continuously transforming substances and energy through the enzyme-catalyzed regulation of biochemical reactions. Strain DGL1 induced the overexpression of plasma membrane ATPase in oat, which was 9.6-fold upregulated at 2 h, 9.5-fold upregulated at 4 h, 8.7-fold upregulated at 8 h, and 21.7-fold upregulated at 12 h. Plasma membrane ATPase, which is located in the cell membrane, provides energy and power for plant nutrient uptake by generating an electrochemical gradient and is involved in regulating a number of important processes in plant growth (Hayakawa et al., 1999). Meanwhile, the phosphoglycerate kinase *PGK* gene was upregulated in all four reciprocal groups. PGK is a key enzyme in the glycolysis and gluconeogenesis processes and provides the energy basis for plant life by catalyzing a phosphate group that produces an ATP molecule (García-Gutiérrez et al., 2018). *PDC2*, the gene encoding pyruvate decarboxylase, was upregulated 14.2-fold at 2 h, 26.2-fold at 4 h, 16.2-fold at 8 h, and 28.1-fold at 12 h. Gluconeogenesis is an important metabolic pathway that produces a precursor to glucose from non-carbohydrates, such as organic acids, fatty acids, amino acids, and glycerol, replenishing the glycogen reserves required for plant growth and development. Therefore, it is hypothesized that DGL1 can facilitate the process of energy metabolism in oats, thus providing more power for growth and development.

#### 3.5.2 Nutrient uptake and metabolism

Nitrogen is a constituent for synthesizing proteins, enzymes, nucleic acids, chlorophyll, and other substances and plays an important role in physiological and biochemical processes, such as photosynthesis and respiration (Loqué et al., 2006). The gene encoding glutamate synthase (GOGAT) was upregulated and expressed in all four interaction groups upon induction by DGL1. Glutamate synthase is a key enzyme in the process of ammonia assimilation in plant nitrogen metabolism, which, together with glutamine synthetase, constitutes the GS/GOGAT cycle and is involved in the formation of nitrogen-containing compounds, such as proteins and nucleic acids, in plants (Labrie et al., 1991). In addition, in higher plants, the AMT ammonium transporter protein is the main pathway mediating the transmembrane transport of ammonium nitrogen from the soil in plant roots (Noh et al., 2001). After DGL1 induction, the ammonium transporter protein encoding the AMT gene on the cell membrane of plant roots was upregulated and expressed in all four reciprocal groups and was upregulated 1.8-fold at 2 h after inoculation, 2.1-fold at 4 h after inoculation, 1.9-fold at 8 h after inoculation, and 2.2-fold at 12 h after inoculation. After the plant absorbs nitrate, it must be reduced to nitrite by nitrate reductase and enter the chloroplast to be degraded by nitrite reductase into ammonium, which can be directly absorbed and utilized by the plant (Stulen and Lanting, 2010). DGL1 upregulated the expression of the nitrate reductase-encoding gene *NR* and nitrite reductase-encoding gene *NiR* in the four treatment groups; therefore, it is presumed that DGL1 promotes the plant's nitrate reduction process, improves the nitrogen utilization efficiency, and promotes plant growth and development.

DGL1 also regulates the ABC transporter protein biosynthetic pathway, including the differential expression of the genes encoding the ABCB1, ABCC1, and ABCG2 proteins. Among them, ABCB1 proteins are closely related to the transport of metabolites, such as IAA, indole-3-propionic acid, and vanillic acid, and can promote the transport of hypocotyl and root tip growth factors in plant roots, regulate the elongation and growth of plant roots, and promote the development of lateral roots and root hairs (Parvathaneni et al., 2013). The ABCC subfamily is involved in the transport of anthocyanins and chlorophyll metabolites and the regulation of ion channels (Sun et al., 2021). The ABCG subfamily is involved in the transport of lipid precursors for cuticle and wax biosynthesis and helps in the synthesis of the plant extracellular barrier (Pighin et al., 2006), thus improving the resistance of plants to stress.

#### 3.5.3 Signal transduction

Strain DGL1 promoted the expression of the growth hormone responsive family gene IAA18, which was upregulated in all four treatment groups (2.0-fold at 2 h, 2.7-fold at 4 h, 3.2-fold at 8 h, and 3.2-fold at 12 h after inoculation). The IAA18 gene has been reported to be involved in the formation of lateral roots in *Arabidopsis* (Ploense et al., 2019). *Cyt b5*, a gene encoding the cytochrome b5 protein, was upregulated 1.8-fold at 2 h, 2.5-fold at 4 h, 3.1-fold at 8 h, and 3.7-fold at 12 h. The cytochrome b5 protein is a heme protein present on the endoplasmic reticulum that participates in various redox reactions in plant cells and regulates the balance of reactive oxygen species (ROS) (Zhang et al., 2015). The gene encoding cytochrome P450 was upregulated 3.6-fold at 2 h, 4.6-fold at 4 h, 4.8-fold at 8 h, and 5.2-fold at 12 h. P450 enzymes are involved in a wide range of plant biosynthetic pathways and play a catalytic role in the biosynthesis of benzene propane, the synthesis of many terpenoids, fatty acid biosynthesis and metabolism, and secondary metabolite production in the plant defense response (Zuo et al., 2021).

#### 3.5.4 Genes related to cell wall formation

*CSLF6*, the gene encoding cellulose synthase, was upregulated 6.0-fold at 2 h, 4.2-fold at 4 h, 3.8-fold at 8 h, and 3.9-fold at 12 h. The *CSL* gene family is widespread in grasses, is involved in the synthesis of plant cellulose, and plays an important role in the synthesis of the secondary cell wall, conferring structural and mechanical support. The gene *CSLF6* is also capable of regulating plant growth and development, and it has been reported that rice *CslF6* mutant plants exhibit a short stature and reduced tiller number (Jin, 2013). The gene encoding 1,3-glucanase was upregulated in all four treatment groups. 1,3-Glucanase is widely distributed in the pollen tube wall, sperm cell wall, and sieve tube wall and is involved in the biosynthesis of the plant cell wall, flower organ development, and plant nutrient transport (Legentil et al., 2006). It is hypothesized that DGL1 can induce the expression of the genes encoding 1,3-glucanases, thus promoting plant cell wall synthesis, organ development, and other physiological processes. Twelve genes encoding cinnamyl alcohol dehydrogenase were differentially expressed, and as a key enzyme in the lignin synthesis pathway, it is able to increase the strength and hydrophobicity of plant cell walls and improve resistance to external environmental conditions (Ma, 2014).

#### 3.5.5 Stress response genes

DGL1 promoted resistance to biotic and abiotic stressors in oats by regulating oat defense and stress response mechanisms. Under DGL1 induction, the gene encoding ethanol dehydrogenase, *ADH3*, was upregulated in all four reciprocal groups. *ADH3* plays an important role in plant resistance to adversity stress, and drought stress was reported to promote the significant expression of *ADH* in tobacco roots. When the *ADH* gene was silenced, tobacco wilted, indicating that the ADH gene can improve plant resistance to adversity stress (Peters and Frenkel, 2004). The gene encoding the C2H2-type zinc finger protein was upregulated 5.0-fold at 2 h, 5.3-fold at 4 h, 5.4-fold at 8 h, and 5.3-fold at 12 h after reciprocal induction with DGL1. The C2H2 zinc finger protein improves plant stress tolerance by increasing the content of the osmoregulatory substances free proline and soluble sugars and by increasing the ability to scavenge ROS, and it is also involved in the growth and development of many plant flowering organs and root hairs (Han et al., 2019). The 26S protease-encoding genes also showed the same upregulated expression, which can regulate a variety of physiological and metabolic processes by participating in plant cell cycle regulation and stress responses (Marshall and Vierstra, 2019).

A total of 121 genes were differentially expressed in response to heat stimulation, including the heat shock protein Hsp20, the heat-stimulated transcription factor HSF, and genes encoding peroxidase isozymes. The Hsps are found in the cytoplasm and nucleus and are essential components for maintaining cellular homeostasis under adverse conditions, such as drought, cold, and high temperature, and Hsp20 acts as a molecular chaperone by binding to partially folded or denatured proteins to prevent the irreversible aggregation of proteins and to maintain stability (Eyles and Gierasch, 2010). Hsf transcription factors not only help organisms resist high temperatures, but also promote seed maturation and root growth (Li et al., 2021). Therefore, it is hypothesized that DGL1 can improve the stability of plant cells in an adversarial environment by inducing the expression of genes during the plant response to thermal stimulation. Meanwhile, DGL1 upregulated the expression of TPR-encoding genes, and TPR proteins have important roles in cell cycle regulation, stress response, gene expression, protein degradation, and other biological processes (Zhou et al., 2022). Lin et al. (2013) found that the *AtTPR1* gene was involved in regulating the growth hormone uptake process in *Arabidopsis*. DGL1 also induced the overexpression of 1,2-oxo-phytodienoic acid reductase (OPR), a key enzyme in the jasmonic acid biosynthetic pathway involved in plant growth, development, hormone, and biotic and abiotic stress-related processes. In wheat, *TaOPR1* transgenic plants increase salinity tolerance in an abscisic acid (ABA)-dependent manner (Beynon et al., 2009).

#### 3.5.6 Transcription factors

After 2, 4, 8, and 12 h of interaction between DGL1 and oats, 133, 223, 221, and 302 differentially expressed transcription factors, respectively, were identified, of which NAC, BhlH, MYB, bZIP, WRK, and C3H were associated with response to plant stress, including seven transcription factors from the MYB family, namely MYB44, MYB88, MYB4, MYB6, MYB3R1, MYB3R2, and MYB3R4. Nine transcription factors from the NAC family, including NAC17, NAC35, NAC53, NAC71, NAC74, NAC78, NAC79, NAC82, and NAC89, were differentially expressed.

**Figure 6:**
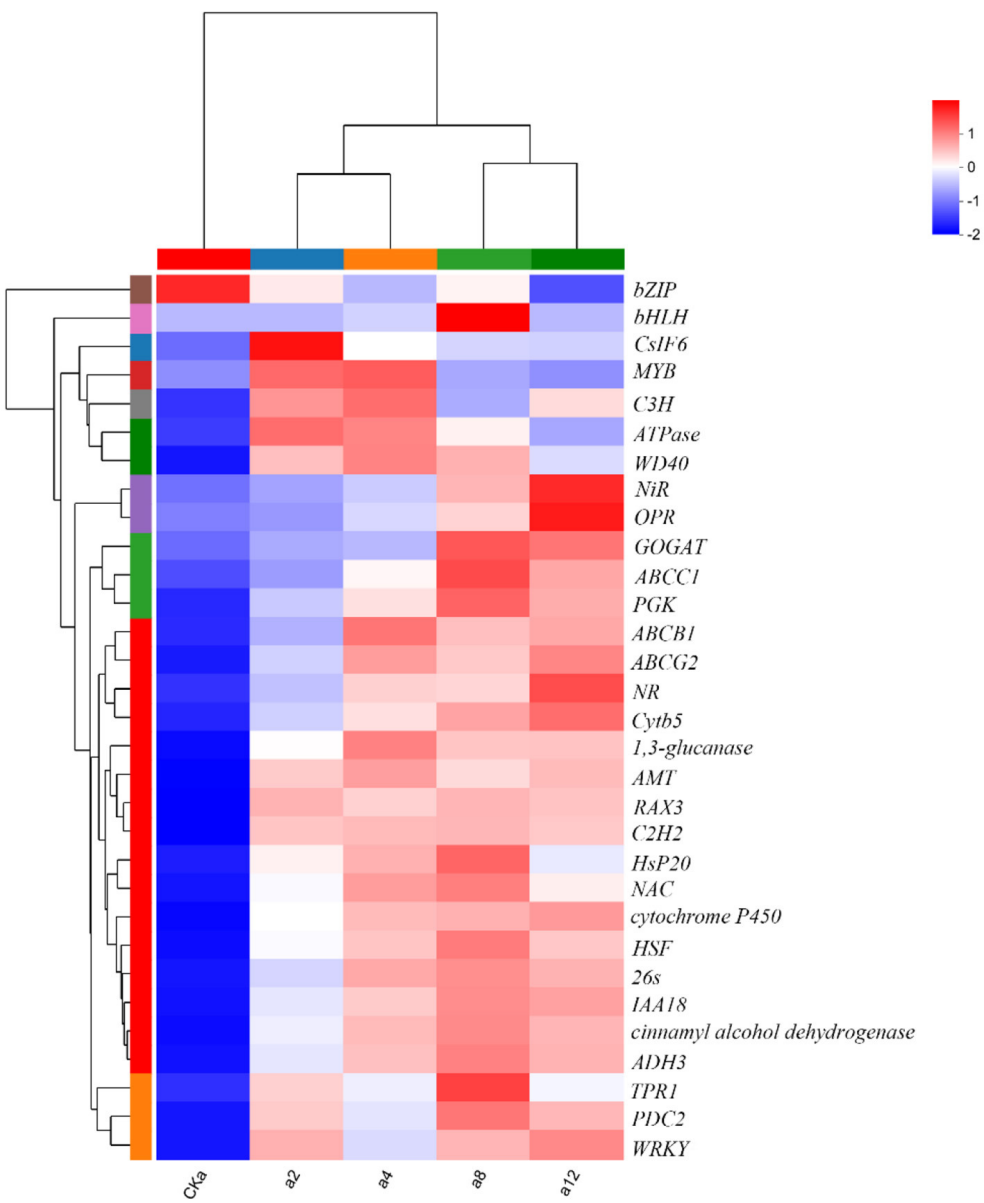
Clustering heatmap of DEGs. Red color represents higher expression of the gene in the sample, blue color represents lower expression, and the closer the two gene branches are to each other, the closer their expression is. The closer the branches of the two samples are, the closer the expression patterns of all genes in these two samples are, and the closer the trends of gene expression changes are.

In summary, DGL1 promoted the expression of genes *ADH3, C2H2*, 26 s, HsP20, *HSF, TPR*, and *OPR1*, which are associated with tolerance to biotic and abiotic stress. Therefore, it is hypothesized that the induced expression of these proteins may increase the adaptive capacity of oats to environmental stress. Genes including *CSLF6*, a 1,3-glucanase-encoding gene, and cinnamyl alcohol dehydrogenase-encoding genes are associated with cell wall synthesis, and it is hypothesized that the induced expression of these genes by DGL1 may increase the mechanical strength of oat cells and enhance oat support and protection functions. Differential expression of *NR*, encoding nitrate reductase, *NiR*, encoding nitrite reductase, glutamate synthase (GOGAT), and the ABC transporter protein may increase the absorption of nutrients from oats and promote the synthesis of proteins, nucleic acids, chlorophyll, and other substances. DGL1 also induced the differential expression of several transcription factors, such as NAC, BhlH, MYB, bZIP, WRK, and C3H, which are involved in regulating gene expression in response to stress (Figure 6).

### 3.6 Real-time fluorescence quantitative PCR validation

**Figure 7:**
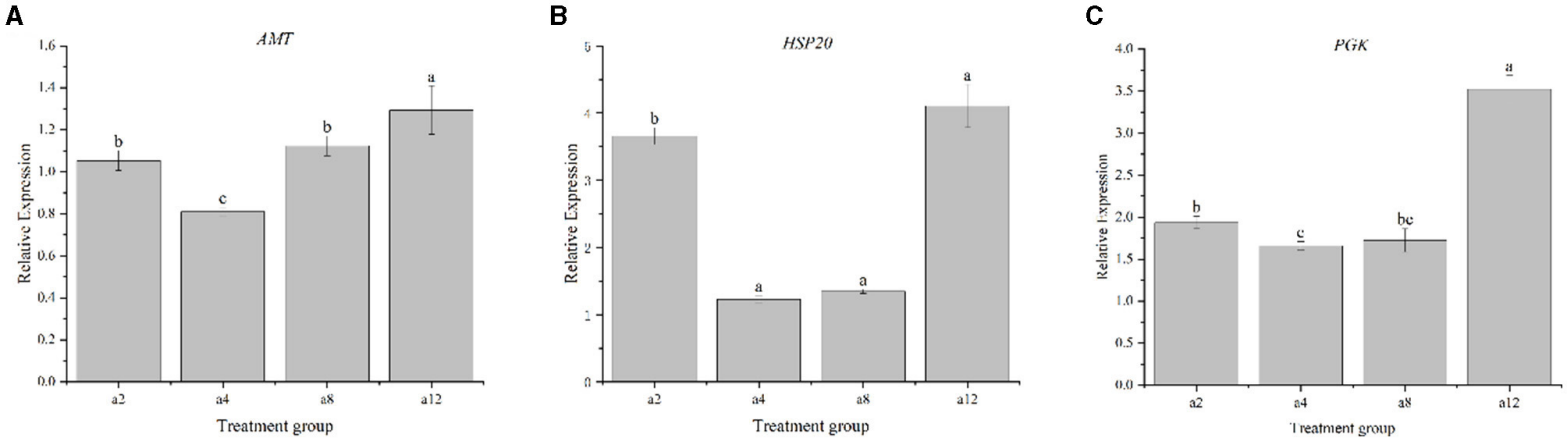
Real-time PCR validation of DEGs. Data represent means ± SD from three biological replicates. Statistically significant differences were determined using multiple comparison (Student–Newman–Keuls test). Different letters indicate a significant difference (*p* < 0.05). **(A)** The gene of AMT, **(B)** The gene of HSP20, and **(C)** The gene of PGK.

The expression patterns of the genes *PGK, AMT*, and *Hsp20* of strain DGL1 at 2, 4, 8, and 12 h after treatment were detected by real-time fluorescence quantitative PCR, and the results showed that (Figure 7) the expression trends of these genes were generally consistent with the RNA-Seq results, indicating that the RNA-Seq data were authentic and reliable.

## 4 Conclusion and discussion

The relationship between plants and inter-root beneficial microorganisms is mutually beneficial, and numerous reports have confirmed that beneficial microorganisms can promote plant growth and increase plant tolerance to abiotic stress, as well as suppress a range of plant diseases caused by pathogenic fungi and bacteria by inducing systemic resistance in plants (Berg, 2015). *Bacillus amyloliquefaciens* DGL1, isolated from the extreme habitats of the Tibetan Plateau, has high-quality biological activity, significantly promotes plant growth, and has antagonistic activity against several phytopathogenic fungi. To investigate the molecular mechanism by which DGL1 promotes growth in oat, transcriptome sequencing was used to analyze the response of the oat root system to this strain following inoculation with DGL1. The differential genes were mainly enriched in pathways affecting nutrient uptake, nitrogen assimilation, metabolism, signal transduction, and defense responses.

*Bacillus* has been reported to be involved in plant growth and development in a variety of ways, including through nitrogen fixation and the regulation of nutrient transporter protein expression to improve nutrient access in host plants (Batista et al., 2021). Transcriptome sequencing analysis revealed that the genes encoding glutamate synthase (GOGAT) and ammonium transporter protein, which exist in the cell membranes of plant roots, and genes encoding nitrate reductase (NR) and nitrite reductase (NiR), which are involved in the uptake, transport, and assimilation of nitrogen in oat, were upregulated in all four reciprocal groups after DGL1 inoculation. In addition, whole-genome sequencing of DGL1 revealed the presence of *glnB*, which acts as an essential gene for nitrogen fixation, and the *NifA* gene plays a regulatory role in symbiotic nitrogen fixation. Therefore, it was hypothesized that DGL1 can enhance nitrogen metabolism in oats, thereby promoting photosynthesis, leaf growth, nutrient uptake, and plant yield and quality.

DGL1 also upregulated the expression of genes encoding *IAA18* in the oat tryptophan metabolic pathway, which binds and interacts with the transcription factor ARF; a transcriptional activator that regulates lateral root formation in *Arabidopsis thaliana*. According to Uehara (2008), mutations in structural domain II of *IAA18* resulted in the inhibition of lateral root formation and affected shoot development in *Arabidopsis*. DGL1 also regulates the ABC transporter protein biosynthetic pathway, including the differential expression of genes encoding the ABCB1 protein, ABCC1 protein, and ABCG2 protein. Among them, the ABCB1 protein and ABCC subfamily proteins are involved in regulating the transport process of plant growth hormones, such as IAA, indole-3-propionic acid, and chlorophyll metabolites, in plant roots, participating in the elongation and growth of plant roots, and promoting the development of lateral roots and root hairs. Meanwhile, in the genomic analysis of DGL1, DGL1 was found to promote key genes for growth hormone synthesis, including *yhcX, dhaS*, and *YsnE*. Therefore, it is hypothesized that DGL1 promotes plant growth by producing plant growth hormones, regulating the expression of growth hormone-related genes, enhancing the transport process of plant hormones by inducing the ABC transporter protein biosynthesis pathway, promoting photosynthesis, and increasing the biomass of oats.

DGL1 also promotes resistance to biotic and abiotic stress in oats by regulating oat defense and stress response mechanisms. The KEGG enrichment analysis revealed that the gene encoding ethanol dehydrogenase, *ADH3*, was upregulated in all four reciprocal groups. Ethanol dehydrogenase is commonly found in plant tissues and is an important hydrolase in plant metabolism. It also has significant regulatory effects on ethanol and acetaldehyde metabolism (Lou et al., 1993). Liang (2013) reported that the expression of the *ADH3* gene was significantly changed when young roots of rice were treated with different NaCl concentrations, which may be a regulatory mechanism by which plants cope with stress. In addition, 121 genes were differentially expressed in response to heat stimulation, including Hsp20, the heat-stimulated transcription factor HSF, and genes encoding peroxidase isozymes. When plants are subjected to stress, such as drought, salt, cold, and high temperature, which adversely affect growth and development, the plant regulatory gene HSP20 prevents protein aggregation, thereby alleviating adversity. *Capsicum annuum* exhibits higher expression levels of HSP20 during heat stress, suggesting its possible involvement in heat stress and defense responses (Guo et al., 2015). The heat stress transcription factor HSF is a major regulator of the plant response to heat stress and is involved in regulating the expression of protective proteins in response to heat stress injury (Schramm et al., 2007). Catalase is an important substance of the antioxidant system in plant tissues that catalyzes the production of excess H_2_O_2_ to generate water and oxygen under plant adversity. Together with superoxide dismutase and peroxidase, it scavenges ROS in plant tissues, thereby eliminating the toxicity of H_2_O_2_ to plant cells and preventing cell damage (Sofo et al., 2015). Therefore, it is hypothesized that DGL1 can induce the expression of genes relevant to the alleviation of adversity stress, thereby improving the resistance of plants to abiotic stress.

The transcription factor encoding the gene *RAX3* was upregulated 1.8-fold at 2 h, 1.8-fold at 4 h, 2.0-fold at 8 h, and 2.1-fold at 12 h. *RAX3* is a MYB-like transcription factor that plays an important role in participating in plant growth and development and regulating biotic and abiotic stress, and silencing *RAX3* in cotton results in reduced water content under drought stress, weakening the resistance to drought stress (Ding et al., 2015). The transcription factor encoding the *WD40* gene was induced by DGL1 and was overexpressed in all four treatment groups and upregulated 2.5-fold at 2 h, 3-fold at 4 h, 2.7-fold at 8 h, and 2.1-fold at 12 h. WD40 forms transcriptional complexes with MYB and bHLH, thus regulating phycocyanin synthesis (Jia et al., 2021). WD40, as a key regulator of developmental and stress signaling, is involved in several plant growth and developmental processes, such as cell division, apoptosis, motility, light signaling, and flowering, in *Arabidopsis* and can enhance the tolerance of *Arabidopsis* to salt and drought stress (Qiu et al., 2021).

Among the genes associated with cell wall formation, the cellulose synthase-encoding gene *CSLF6* was highly upregulated in all four reciprocal groups, and the CSL gene plays an important role in secondary cell wall synthesis. According to Vega-Sánchez et al. (2015), the *CslF6* gene is required for the accumulation of mixed glycoside-bonded glucans in the non-lignified primary cell wall of *Oryza sativa*. The CslF6 gene is important for the synthesis of (1,3, 1,4)-β-D-glucan. In addition, the content of (1,3, 1,4)-β-D-glucan in the oat cells reached 70–80%, which not only provides mechanical support to the plant cells but also serves as a nutrient store to some of the components needed by the cells. In *O. sativa*, the *OsCslF6* knockout mutant led to a reduction in the amount of glucan synthesized in rice, as reported by Taketa et al. (2012). Of the oat secondary metabolites, cinnamyl alcohol dehydrogenase, a key enzyme in the lignin synthesis pathway, adds strength and hydrophobicity to plant cell walls and resists invasion of plant tissues by pathogenic bacteria. Transgenic tomato plants overexpressing CAD2, the gene encoding cinnamyl alcohol dehydrogenase, have been reported to possess greater growth vigor. In addition, these transgenic tomato plants contain a higher lignin content and CAD enzyme activity in the stem, leaf, and pericarp tissues compared with wild-type tomato plants (Li et al., 2019).

In summary, the molecular mechanism by which *Bacillus* DGL1 acts on oat growth was investigated by RNA-Seq analysis of DEGs and related metabolic pathways in the oat root system at different times after inoculation. It is hypothesized that the growth-promoting mechanism of DGL1 in oats is the result of the coordination of multiple pathways, including the promotion of oat energy metabolism, phytohormone signaling, secondary metabolite synthesis, and amino acid metabolism. This study provides a theoretical basis for the promotion of oat growth by inter-root growth-promoting bacteria in extreme habitats of the Tibetan Plateau.

## Data availability statement

The original contributions presented in the study are included in the article/Supplementary material, further inquiries can be directed to the corresponding author.

## Author contributions

XY: Data curation, Methodology, Writing – original draft, Writing – review & editing. YX: Funding acquisition, Project administration, Resources, Writing – review & editing. TW: Supervision, Writing – review & editing. YQ: Resources, Writing – review & editing. JL: Supervision, Writing – review & editing. LW: Supervision, Writing – review & editing. YG: Supervision, Writing – review & editing.

## References

[B1] Batista B. D. Dourado M. N. Figueredo E. F. Hortencio R. O. Marques J. Piotto F.. (2021). The auxin-producing *Bacillus thuringiensis* RZ2MS9 promotes the growth and modifies the root architecture of tomato (Solanum lycopersicum cv. Micro-Tom). Arch. Microbiol. 203, 3869–3882. 10.1007/s00203-021-02361-z 34013419

[B2] Berg G. (2015). Plant-microbe interactions promoting plant growth and health: perspectives for controlled use of microorganisms in agriculture. Appl. Microbiol. Biotechnol. 84, 11–18. 10.1007/s00253-009-2092-7 19568745

[B3] Beynon E. R. Symons Z. C. Jackson R. G. Lorenz A. Rylott E. L. Bruce N. C. (2009). The role of oxophytodienoate reductases in the detoxification of the explosive 2,4,6-trinitrotoluene by Arabidopsis. Plant Physiol. 151,253–261. 10.1104/pp.109.141598 19605548 PMC2735992

[B4] Chen Z. W. Wang L. Niu R. (2018). Distribution of β-glucan and phenolic acids in bran obtained from the processing of naked oat rice and oat flour. Food Sci. 39, 1–6. 10.1016/j.jfoodeng.2017.11.002

[B5] Choudhary D. K. Johri B. N. (2008). Interactions of *Bacillus* spp. and plants–with special reference to induced systemic resistance (ISR). Microbiol. Res. 64, 493–513. 10.1016/j.micres.2008.08.007 18845426

[B6] Ding Z. Q. Chen T. Z. Liu T. L. (2015). Functional analysis of drought-induced MYB-like transcription factor GhRAX3 in cotton. Chin. Agricult. Sci. 18,12–21. 10.3864/j.issn.0578-1752.2015.18.001

[B7] Elshaghabee F. Rokana N. Gulhane R. D. Sharma C. Panwar H. (2017). *Bacillus* as potential probiotics: status, concerns, and future perspectives. Front. Microbiol. 10, 1490. 10.3389/fmicb.2017.01490 28848511 PMC5554123

[B8] Eyles S. J. Gierasch L. M. (2010). Nature's molecular sponges: small heat shock proteins grow into their chaperone roles. Proc. Natl. Acad. Sci. U S A. 107, 2727–2728. 10.1073/pnas.0915160107 20133678 PMC2840345

[B9] García-Gutiérrez Á. Cánovas F. M. Ávila C. (2018). Glutamate synthases from conifers: gene structure and phylogenetic studies. BMC Genomics. 19, 65. 10.1186/s12864-018-4454-y 29351733 PMC5775586

[B10] Gene O. C. (2010). The Gene Ontology in 2010: extensions and refinements. Nucleic Acids Res. 38, 331–335. 10.1093/nar/gkp1018 19920128 PMC2808930

[B11] Guan X. Jin S. Li S. Huang K. Liu J. (2018). Process optimization, characterization and antioxidant capacity of oat (*Avena Sativa* L.) bran oil extracted by subcritical butane extraction. Molecules 23, 1546. 10.3390/molecules23071546 29954066 PMC6099595

[B12] Guo M. Liu J. H. Lu J. P. Zhai Y. F. Wang H. Gong Z. H.. (2015). Genome-wide analysis of the CaHsp20 gene family in pepper: comprehensive sequence and expression profile analysis under heat stress. Front. Plant Sci. 6, 806. 10.3389/fpls.2015.00806 26483820 PMC4589653

[B13] Guo W. Tzioutziou N. Stephen G. Milne I. Calixto C. P. Waugh R.. (2020). 3D RNA-seq: a powerful and flexible tool for rapid and accurate differential expression and alternative splicing analysis of RNA-seq data for biologists. RNA Biol. 18, 1574–1587. 10.1080/15476286.2020.1858253 33345702 PMC8594885

[B14] Han G. Yuan F. Guo J. Zhang Y. Sui N. Wang B. (2019). AtSIZ1 improves salt tolerance by maintaining ionic homeostasis and osmotic balance in Arabidopsis. Plant Sci. 285, 55–67. 10.1016/j.plantsci.2019.05.002 31203894

[B15] Hayakawa T. Hopkins L. Peat L. J. Yamaya T. Tobin A. K. (1999). Quantitative intercellular localization of NADH-dependent glutamate synthase protein in different types of root cells in rice plants. Plant Physiol. 119, 409–416. 10.1104/pp.119.2.409 9952435 PMC32116

[B16] Jia N. Wang J. Wang Y. Ye W. Liu J. Jiang J.. (2021). The light-induced WD40-repeat transcription factor DcTTG1 regulates anthocyanin biosynthesis in Dendrobium candidum. Front. Plant 17, 12–63. 10.3389/fpls.2021.633333 33815441 PMC8010245

[B17] Jin C. (2013). Functional study of rice cellulase-like gene *OsCSLF. Huazhong Agricult. Univer*. 22–27. 10.7666/d.Y2394732

[B18] Kanehisa M. Goto S. (2011). KEGG: kyoto encyclopedia of genes and genomes. Nucleic Acids Res. 28, 27–30. 10.1093/nar/28.1.27 PMC102409 10592173

[B19] Łabanowska M. Kurdziel M. Filek M. Wesełucha-Birczyńska A. (2016). The impact of biochemical composition and nature of paramagnetic species in grains on stress tolerance of oat cultivars. J. Plant Physiol. 199, 52–66. 10.1016/j.jplph.2016.04.012 27302006

[B20] Labrie S. T. Wilkinson J. Q. Crawford N. M. (1991). Effect of chlorate treatment on nitrate reductase and nitrite reductase gene expression in arabidopsis thaliana. Plant Physiol. 97, 873–879. 10.1104/pp.97.3.873 16668525 PMC1081098

[B21] Legentil L. Paris F. Ballet C. Trouvelot S. Daire X. Vetvicka V.. (2006). Molecular interactions of β-(1-3)-glucans with their receptors. Molecules. 20, 9745–9766. 10.3390/molecules20069745 PMC6272582 26023937

[B22] Li M. Cheng C. Zhang X. Zhou S. Li L. Yang S. (2019). Overexpression of pear (*Pyrus pyrifolia*) CAD2 in tomato affects lignin content. Molecules 24, 2595. 10.3390/molecules24142595 31319487 PMC6680972

[B23] Li X. T. Feng X. Y. Zeng Z. Liu Y. Shao Z. Q. (2021). Comparative analysis of HSF genes from secale cereale and its triticeae relatives reveal ancient and recent gene expansions. Front. Genet. 12, 8012–8018. 10.3389/fgene.2021.801218 34887907 PMC8650501

[B24] Liang Y. (2013). Comparison of ethanol dehydrogenase 3 expression in different salt-sensitive rice young roots under salt stress. Jiangsu Agricult. Sci. 41, 33–35. 10.15889/j.issn.1002-1302.2013.11.093

[B25] Lin Z. Ho C. W. Grierson D. (2013). AtTRP1 encodes a novel TPR protein that interacts with the ethylene receptor ERS1 and modulates development in Arabidopsis. J. Exp. Bot. 60, 3697–3699. 10.1093/jxb/erp209 19567478 PMC2736885

[B26] Liu J. X. Wang J. C. Wang R. J. Jia H. Y. (2017). Effect of complex saline-alkali stress on the mineral ions absorption and photosynthetic characteristics of oat seedlings. Agric. Res. Arid Areas 35,178–184. 10.7606/j.issn.1000-7601.2017.01.27

[B27] Loqué D. Yuan L. Kojima S. Gojon A. Wirth J. Gazzarrini S.. (2006). Additive contribution of AMT1;1 and AMT1;3 to high-affinity ammonium uptake across the plasma membrane of nitrogen-deficient Arabidopsis roots. Plant J. 48, 522–534. 10.1111/j.1365-313X.2006.02887.x 17026539

[B28] Lou H. McCullough A. J. Schuler M. A. (1993). Expression of maize Adh1 intron mutants in tobacco nuclei. Plant J. 3, 393–403. 10.1046/j.1365-313X.1993.t01-22-00999.x 8220449

[B29] Ma Q. H. (2014). Functional analysis of a cinnamyl alcohol dehydrogenase involved in lignin biosynthesis in wheat. J. Exp. Bot. 61, 2735–2744. 10.1093/jxb/erq107 20400532 PMC2882267

[B30] Marshall R. S. Vierstra R. D. (2019). Dynamic regulation of the 26S proteasome: from synthesis to degradation. Front Mol Biosci. 2, 285. 10.3389/fmolb.2019.00040 31231659 PMC6568242

[B31] Mustafa G. (2011). Nitrogen and irrigation effects on grain β-glucan content of oats (*Avena sativa* L.). Aust. J. Crop Sci. 5, 242–247.

[B32] Nan M. Zhao G. Q. LI J. (2018). Relationship between morphological characteristics of different oat varieties and their resistance to buckling. J. Grassl. 26, 1382–1391.

[B33] Noh B. Murphy A. S. Spalding E. P. (2001). Multidrug resistance-like genes of Arabidopsis required for auxin transport and auxin-mediated development. Plant Cell. 13, 2441–2454. 10.1105/tpc.010350 11701880 PMC139463

[B34] Ortiz A. Sansinenea E. (2018). Chemical compounds produced by *Bacillus* sp. factories and their role in nature. Mini. Rev. Med. Chem. 19, 373–380. 10.2174/1389557518666180829113612 30156158

[B35] Palmgren M. Morsomme P. (2020). The plasma membrane H+ -ATPase, a simple polypeptide with a long history. Yeast. 36, 201–210. 10.1002/yea.3365 30447028 PMC6590192

[B36] Parvathaneni R. Spiekerman J. Zhou H. Wu X. Devos K. (2013). Structural characterization of ABCB1, the gene underlying the d2 dwarf phenotype in pearl millet. cenchrus americanus (L.) morrone. G3 (Bethesda). 9, 2497-2509. 10.1534/g3.118.200846 PMC6686935 31208958

[B37] Peters J. Frenkel C. (2004). Relationship between alcohol dehydrogenase activity and low-temperature in two maize genotypes, Silverado F.and AdhI - Adh2 - doubly null. Plant Physiol. Biocth. 42, 841–846. 10.1016/j.plaphy.2004.10.004 15596104

[B38] Pighin J. A. Zheng H. Balakshin L. J. Goodman I. P. Western T. L. Jetter R.. (2006). (2004). Plant cuticular lipid export requires an ABC transporter. Science 306, 702–704. 10.1126/science.1102331 15499022

[B39] Ploense S. E. Wu M. F. Nagpal P. Reed J. W. (2019). A gain-of-function mutation in IAA18 alters Arabidopsis embryonic apical patterning. Development. 136, 1509–1517. 10.1242/dev.025932 19363152 PMC2674258

[B40] Qiu Y. Tao R. Feng Y. Xiao Z. Zhang D. Peng Y.. (2021). EIN3 and RSL4 interfere with an MYB-bHLH-WD40 complex to mediate ethylene-induced ectopic root hair formation in *Arabidopsis*. Proc. Natl. Acad. 118,2110004118. 10.1073/pnas.2110004118 34916289 PMC8713768

[B41] Radhakrishnan R. Hashem A. Abd A. E. (2017). *Bacillus*: a biological tool for crop improvement through bio-molecular changes in adverse environments. Front. Physiol. 6, 667. 10.3389/fphys.2017.00667 28932199 PMC5592640

[B42] Rojas-Pirela M. Andrade-Alviárez D. Rojas V. Kemmerling U. Cáceres A. J. Michels P. A.. (2003). Phosphoglycerate kinase: structural aspects and functions, with special emphasis on the enzyme from Kinetoplastea. Open Biol. 10, 200302. 10.1098/rsob.200302 33234025 PMC7729029

[B43] Saeed Q. Xiukang W. Haider F. U. Kučerik J. Mumtaz M. Z. Holatko J.. (2021). Rhizosphere bacteria in plant growth promotion, biocontrol, and bioremediation of contaminated sites: a comprehensive review of effects and mechanisms. Int. J. Mol. Sci. 22, 105–129. 10.3390/ijms221910529 34638870 PMC8509026

[B44] Schramm F. Larkindale J. Kiehlmann E. (2007). A cascade of transcription factor DREB2A and heat stress transcription factor HsfA3 regulates the heat stress response of Arabidopsis. Plant J. 53, 264–274. 10.1111/j.1365-313X.2007.03334.x 17999647

[B45] Sofo A. Scopa A. Nuzzaci M. Vitti A. (2015). Ascorbate peroxidase and catalase activities and their genetic regulation in plants subjected to drought and salinity stresses. Int. J. Mol. Sci. 16, 13561–13578. 10.3390/ijms160613561 26075872 PMC4490509

[B46] Stulen I. Lanting L. (2010). Nitrate reductase and nitrite reductase activity in dark rown radish seedlings: relation to the supply of NADPH. Physiologia Plantarum. 37, 139–142. 10.1111/j.1399-3054.1976.tb03947.x

[B47] Sun N. Xie Y. F. Wu Y. Guo N. Li D. H. Gao J. (2021). Genome-wide identification of *ABCC* gene family and their expression analysis in pigment deposition of fiber in brown cotton (*Gossypium hirsutum*). PLoS ONE. 16. 0246649. 10.1371/journal.pone.0246649 33961624 PMC8104370

[B48] Taketa S. You T. Tonooka T. Tsumuraya Y. Inagaki Y. Haruyama N.. (2012). Functional characterization of barley betaglucanless mutants demonstrates a unique role for CslF6 in (1,3;1,4)-β-D-glucan biosynthesis. J. Exp. Bot. 63, 381–392. 10.1093/jxb/err285 21940720 PMC3245474

[B49] Uehara T. (2008). Domain II mutations in CRANE/IAA18 suppress lateral root formation and affect shoot development in Arabidopsis thaliana. Plant Cell Physiol. 49, 1025–1038. 10.1093/pcp/pcn079 18505759

[B50] Vega-Sánchez M. E. Verhertbruggen Y. Christensen U. (2015). Loss of Cellulose synthase-like F6 function affects mixed-linkage glucan deposition, cell wall mechanical properties, and defense responses in vegetative tissues of rice. Plant Physiol. 159, 56–69. 10.1104/pp.112.195495 22388489 PMC3375985

[B51] Xie Y. L. Renato D. Stefania M. (2017). Molecular identification of several strains of cellulose-solubilizing *Bacillu*s sp. and their anticorrosive and growth-promoting properties. Microbiol. Bullet. 44, 348–357. 10.13344/j.microbiol.china.160099

[B52] Xu M. Sun Y. Chen H. (2022). Progress in the application of reciprocal transcriptome sequencing technology in microbial research. J. Sichuan Univers. 53, 201–207. 10.12182/20220360203 PMC10409366 35332718

[B53] Yang X. Wang T. Xie Y. Qiao Y. Chen H. Chen L.. (2022). Molecular mechanism and metabolic pathway of *Bacillus amyloliqueque-bacillus* DGL1 promoting oat growth. J. Grassland Sci. 30, 11–18. 10.11733/j.issn.1007-0435.2022.11.007

[B54] Yang X. Xie Y. L. Chen L. (2020). Forage-promoting activity of *Bacillus amyloliquefaciens* DGL1 in the inter-root of white spurge in Qinghai sandy land and its genomic analysis. J. Grasslands. 29, 1637–1648. 10.11733/j.issn.1007-0435.2021.08.005

[B55] Zhang H. Myshkin E. Waskell L. (2015). Role of cytochrome b5 in catalysis by cytochrome P450 2B4. Biochem. Biophys. Res. Commun. 338, 499–506. 10.1016/j.bbrc.2005.09.022 16182240

[B56] Zhou X. Zheng Y. Cai Z. Wang X. Liu Y. Yu A.. (2022). Identification and functional analysis of tomato TPR gene family. Int. J. Mol. Sci. 22, 758. 10.3390/ijms22020758 33451131 PMC7828616

[B57] Zuo H. L. Huang H. Y. Lin Y. C. Cai X. Kong X. Luo D.. (2021). Enzyme activity of natural products on cytochrome P450. Molecules. 27, 515. 10.3390/molecules27020515 35056827 PMC8779343

