# Supplementary figures and images for: Transcriptomic analysis of the response of Avena sativa to Bacillus amyloliquefaciens DGL1
Source: Front Microbiol. 2024 Apr 3;15:1321989. doi: 10.3389/fmicb.2024.1321989 (PMC11022965; doi:10.3389/fmicb.2024.1321989)

# Venn

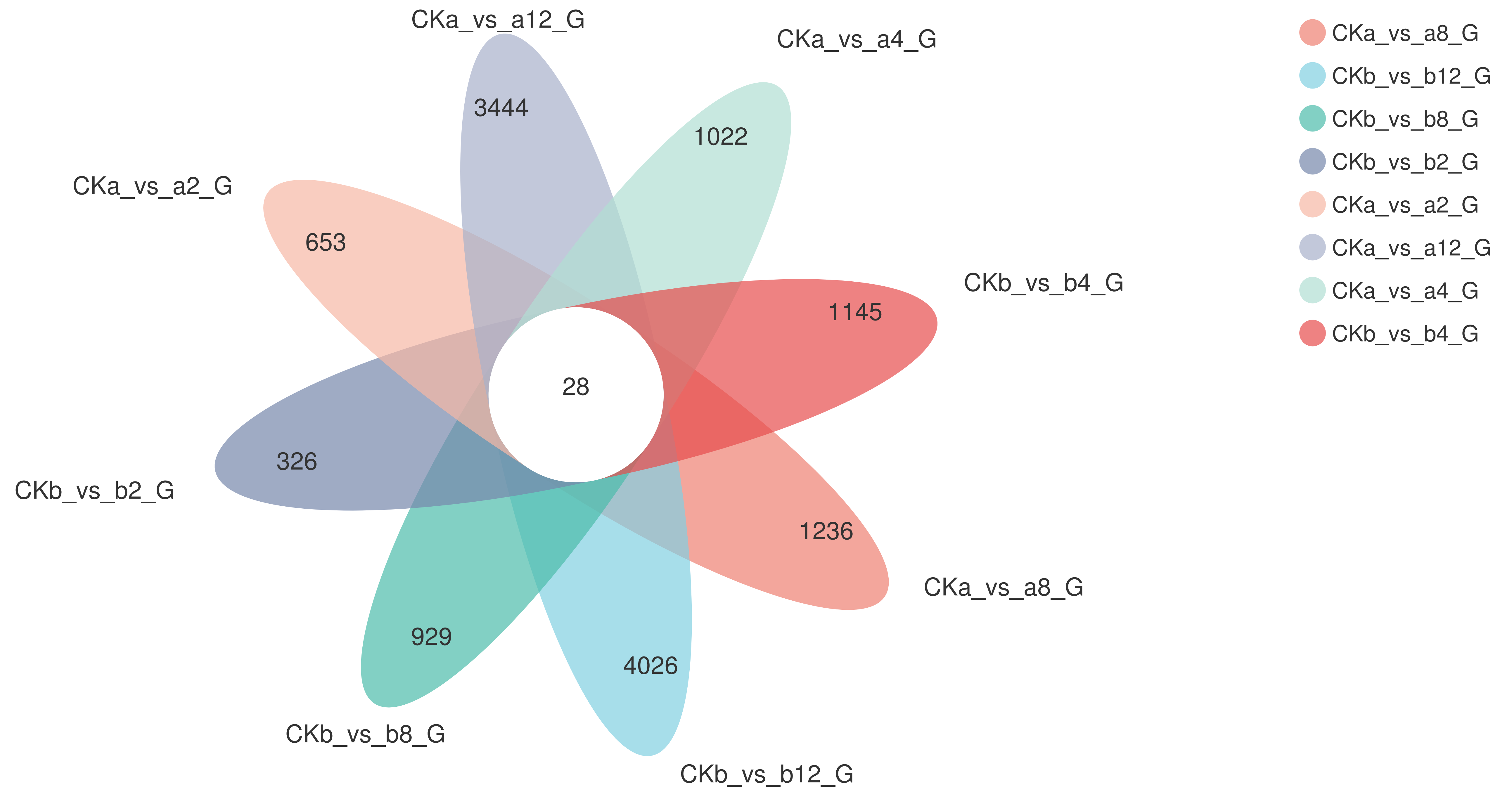

Supplement: Supplementary file 2 [file Data_Sheet_1.PDF]
