# Supplementary material for: Transcriptomic analysis of the response of Avena sativa to Bacillus amyloliquefaciens DGL1
Source: Front Microbiol. 2024 Apr 3;15:1321989. doi: 10.3389/fmicb.2024.1321989 (PMC11022965; doi:10.3389/fmicb.2024.1321989)
Supplement: Supplementary file 3 [file Image_1.pdf]

Supplementary Material

Supplementary Figures

A. Figure 1

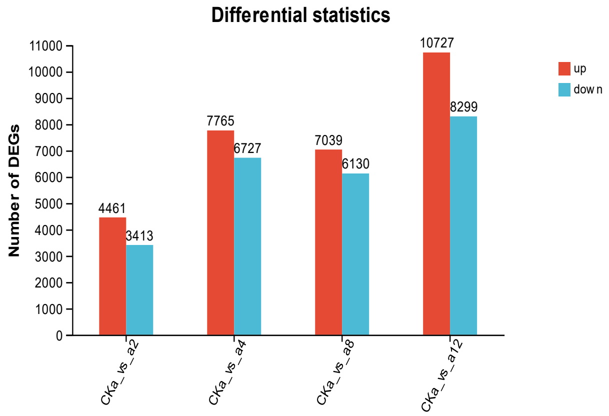

Fig. 1 Statistics of Differentially Expressed Genes

Note: The horizontal coordinates represent four different differently comparison groups, CKa vs a2, CKa vs a4, CKa vs a8, and CKa vs a12, respectively, and the vertical coordinates represent the corresponding number of up and down regulated genes, with red representing up-regulation and blue representing down-regulation.

B. Figure 2 (A、B、C、D)

(A)

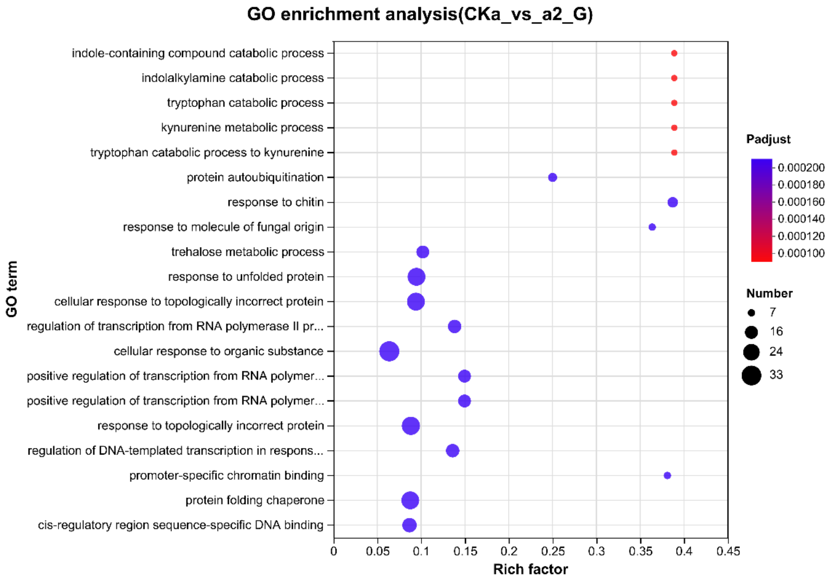

(B)

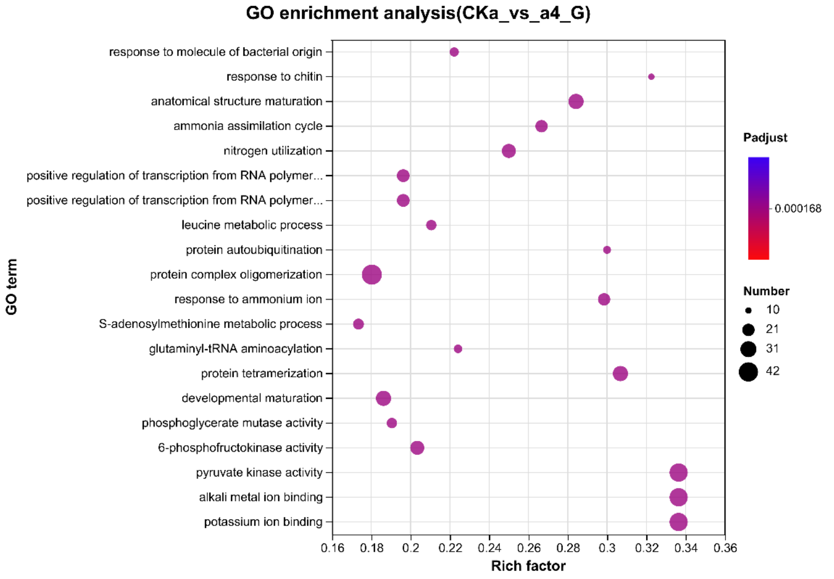

(C)

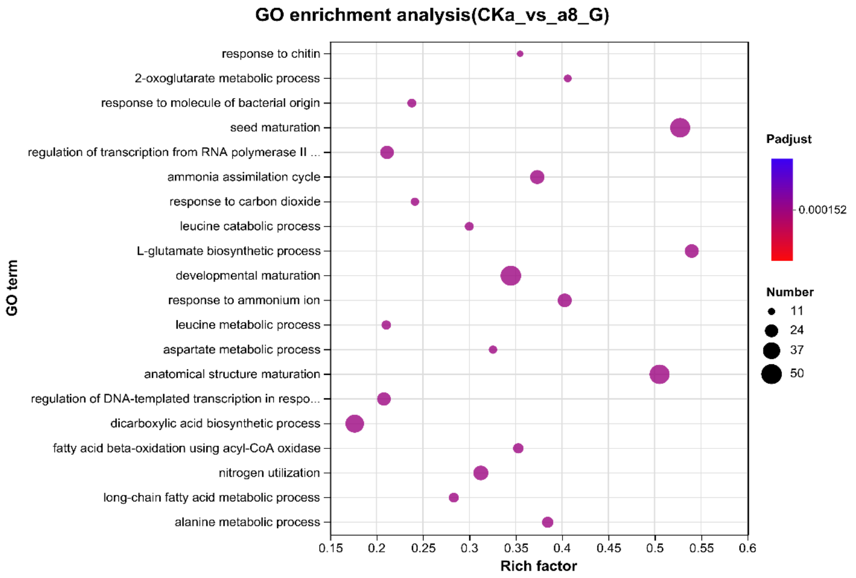

(D)

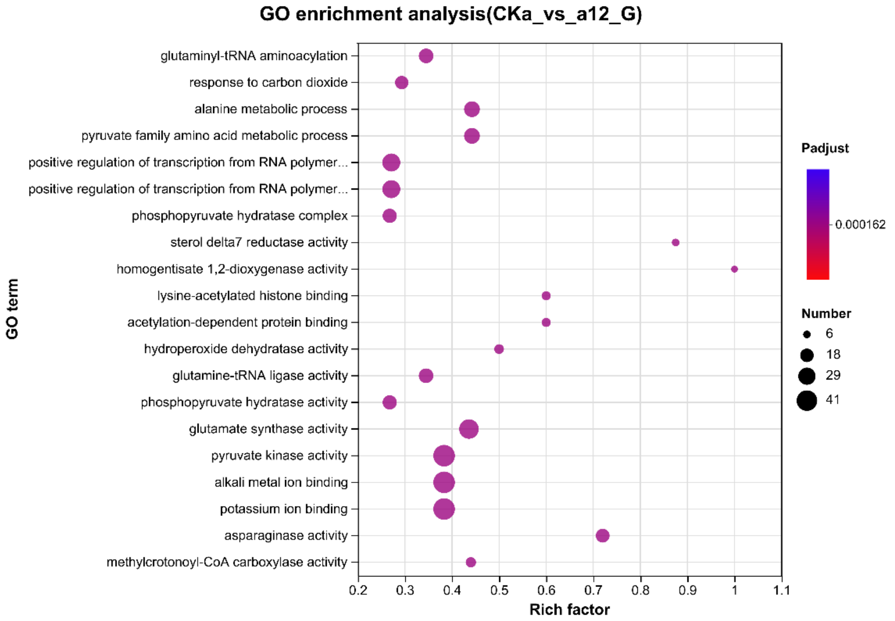

Fig. 2 Classification of Gene Ontology (GO) Function

Note: A:2 h difference comparison group GO functional classification, B:4 h difference comparison group GO functional classification, C:8 h difference comparison group GO functional classification, D:12 h difference comparison group GO functional classification, the vertical axis indicates GO Term, the horizontal axis indicates Rich factor, the size of the dots indicates the number of genes/transcripts in this GO Term, and the color of the dots corresponds to different Padjust ranges.

C. Figure 3 (A、B、C、D)

（A）

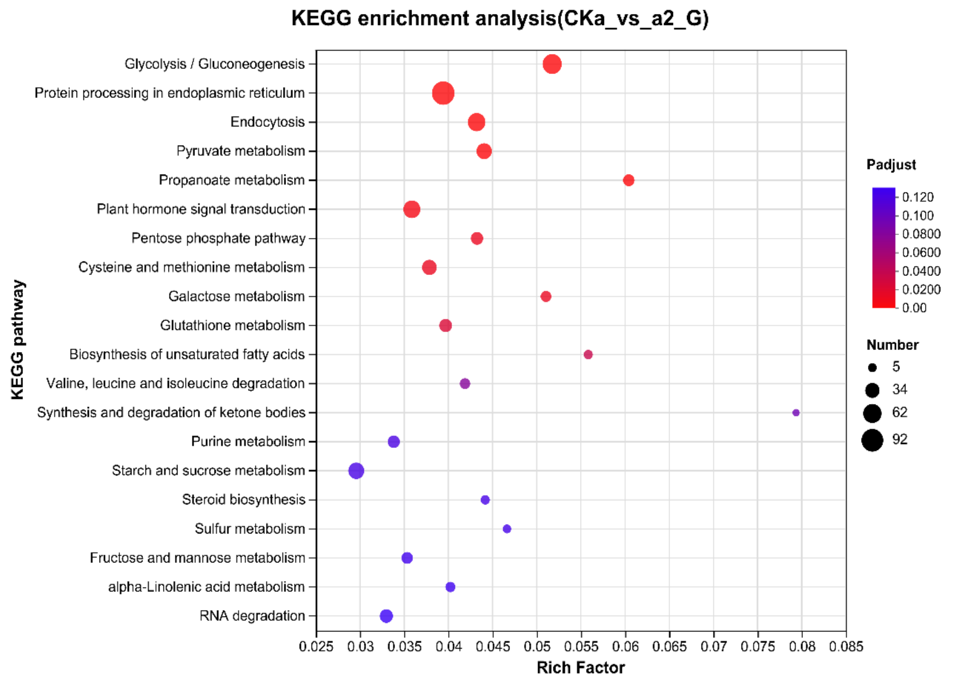

(B)

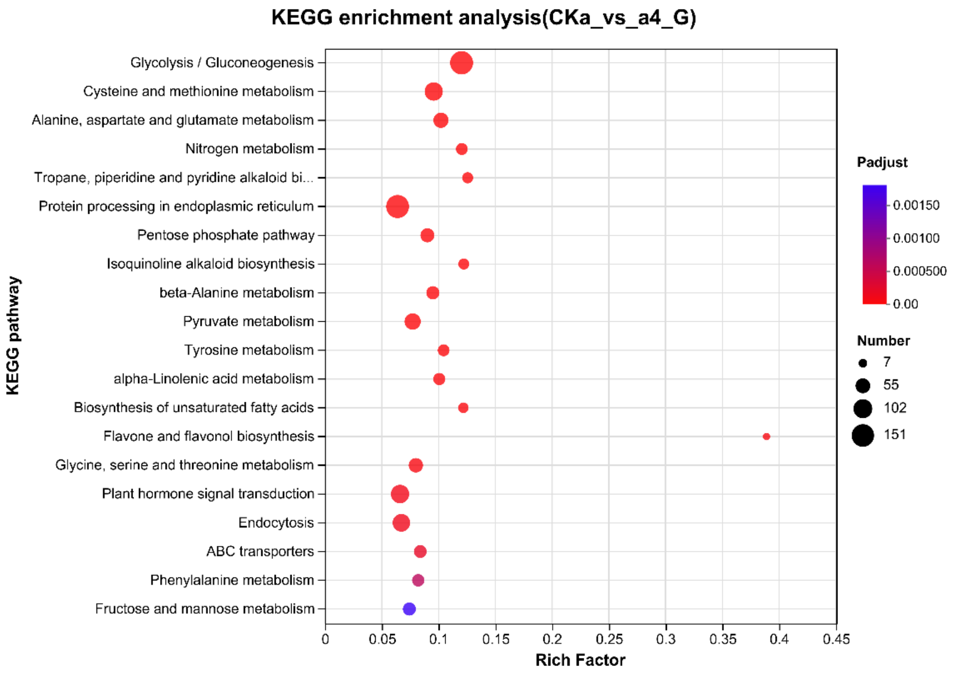

(C)

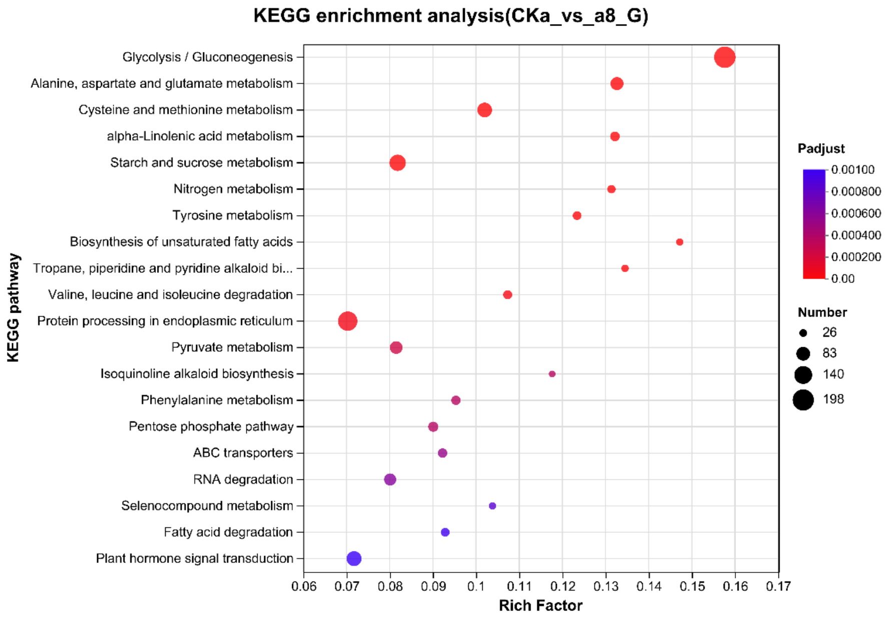

(D)

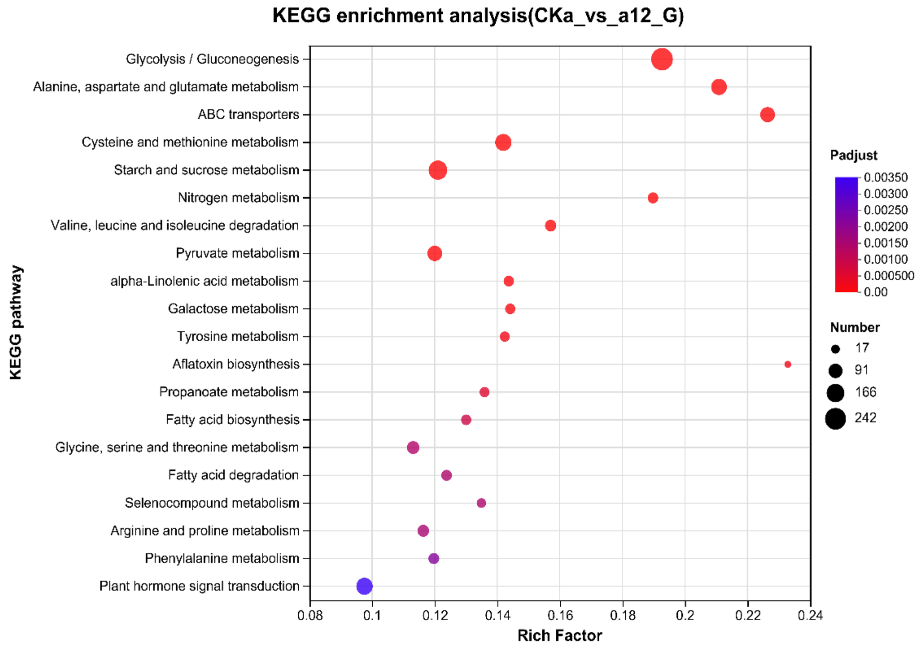

Fig. 3 Enriched Kyoto Encyclopedia of Genes and Genomes (KEGG) Pathways

Note: A:2 h difference comparison group KEGG functional classification, B:4 h difference comparison group KEGG functional classification, C:8 h difference comparison group KEGG functional classification, D:12 h difference comparison group KEGG functional classification, the vertical axis represents the name of the pathway, the horizontal axis represents the Rich factor, the size of the dots indicates the number of genes in this pathway, and the color of the dots corresponds to different padjust ranges.

D. **Figure 4**

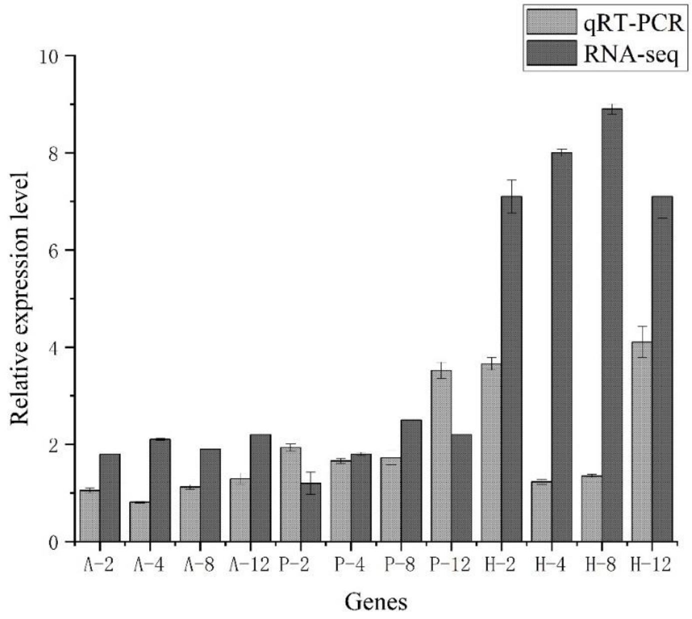

Fig. 4 Validation of Gene Data by qRT-PCR

Note: A-2, A-4, A-8 and A-12 denote the *AMT* gene in the 2, 4, 8 and 12 h treatment groups, respectively; P-2, P-4, P-8 and P-12 denote the PGK gene in the 2, 4, 8 and 12 h treatment groups, respectively; H-2, H-4, H-8 and H-12 denote the Hsp202h gene in the 2, 4, 8 and 12 h treatment groups, respectively;
